# Interfacial Bonding Improvement through Nickel Decoration on Carbon Nanotubes in Carbon Nanotubes/Cu Composite Foams Reinforced Copper Matrix Composites

**DOI:** 10.3390/nano12152548

**Published:** 2022-07-25

**Authors:** Dan Wang, An Yan, Yichun Liu, Zhong Wu, Xueping Gan, Fengxian Li, Jingmei Tao, Caiju Li, Jianhong Yi

**Affiliations:** 1 School of Materials Science and Engineering, Kunming University of Science and Technology, Kunming 650093, China; doromerd@163.com (D.W.); kustya@163.com (A.Y.); clfxl@kust.edu.cn (F.L.); kiwimaya@126.com (J.T.); lilcj@kust.edu.cn (C.L.); yijianhong@kmust.edu.cn (J.Y.); 2 School of Materials Science and Engineering, Tianjin University, Tianjin 300072, China; 3 State Key Laboratory of Powder Metallurgy, Central South University, Changsha 410083, China; ganxueping@csu.edu.cn

**Keywords:** carbon nanotubes, copper matrix composite, three-dimensional reinforcement, interface modification, electrical properties, mechanical properties

## Abstract

Inhomogeneous structures with carbon nanotubes (CNTs), reinforced with Cu composite foams as the reinforcing skeletons (CNTs/Cu_f_^®^Cu), have been designed to overcome the paradox between strength and ductility or conductivity in copper matrix composites. The interface between CNTs and the copper matrix is usually weak, due to poor wettability and interaction. In this study, nickel nanoparticles are decorated onto CNTs to improve interfacial bonding. The broader interface transition area between CNTs and copper with Ni_3_C interfacial products formed, and a combination of improved electrical conductivity (95.6% IACS), tensile strength (364.9 MPa), and elongation (40.6%) was achieved for the Ni-decorated CNTs/Cu_f_^®^Cu (Ni-CNTs/Cu_f_^®^Cu). In addition, the strengthening mechanisms are discussed in this study.

## 1. Introduction

With the possibility of combining outstanding mechanical performance, electrical/thermal conductivity, and tribological properties, CNTs/Cu composites are considered promising candidates for electrical contacting, electrical packaging, and heat exchanging materials [1,2]. Most research has aimed at generating a homogeneous distribution of CNTs throughout the matrix volume, using methods of high-energy ball milling, in situ chemical vapor deposition, friction stir processing, etc. [3,4,5,6,7]. However, dispersing CNTs throughout the entire matrix volume results in composite interface distribution throughout the entire composite volume, which significantly influences the dislocation motion and electron transporting and results in reduced plasticity and conductivity; thus, ductility and conductivity are usually sacrificed in favor of enhanced strength [8,9,10,11,12,13,14,15,16].

In our previous work, we have designed and prepared a copper matrix composite reinforced by CNTs/Cu composite foams, which act as three-dimensional skeletons to enhance the composite strength and tribological properties, and the pure copper phase continuously filling in the foam holes allows the composite to maintain good ductility and conductivity [17,18]. However, problems of poor wettability and interfacial compatibility exist between CNTs and Cu; these problems are believed to be other key factors limiting the performance of CNTs/Cu composites [19,20]. As reported, the addition of carbide-forming elements such as Ti, Cr, and Ni helps form chemical bonds between CNTs and copper, allowing the load to be transferred from the metal matrix to the reinforcements more effectively, and thereby improving the mechanical properties of the composites [21,22,23,24]. In this study, nickel nanoparticles were uniformly modified on the surface of CNTs by an ultrasonic-assisted electroless nickel plating process (abbreviated as Ni-CNTs). Ni-CNTs will be employed in the CNTs/Cu composite foams (abbreviated as Ni-CNTs/Cu_f_) and in the three-dimensional skeleton-reinforced composites (abbreviated as Ni-CNTs/Cu_f_^®^Cu) to improve the interfacial bonding and properties. The microstructure, electrical conductivity, mechanical properties, and strengthening mechanism will be studied and discussed.

## 2. Experimental

### 2.1. Electroless Plating of Nickel on CNTs

First, pristine CNTs (Chengdu Institute of Organic Chemistry Co., Ltd., China) with an inner diameter of 5–20 nm, outer diameter of 30–50 nm, and average length of 10 µm were pretreated. Before electroless plating, these pretreatments include acidification, sensitization, and activation. First, the CNTs were added to a mixed solution of concentrated H_2_SO_4_/HNO_3_ (volume ratio 3:1) for acidification for 4 h and then washed for neutrality. Then, the acidified CNTs were added to SnCl_2_∙2H_2_O (10 g/L) and HCl (40 g/L) solution for sonication for 30 min, filtered, and dried. Finally, the sensitized CNTs were added to a solution of PbCl_2_ (1 g/L) and HCl (100 mL/L) for 30 min under ultrasonic wave, washed, and dried to obtain pretreated CNTs. After pretreatment, the CNTs were immersed in an electroless nickel plating solution at 40 °C for ultrasonic reaction over 30 min. The formula of the plating solution comprised NiSO_4_·6H_2_O (28 g/L), NaH_2_PO_2_·H_2_O (28 g/L), Na_3_C_6_H_5_O_7_·2H_2_O (16 g/L), and CH_3_COONa (16 g/L). The pH value of the plating solution was maintained between 4.5 and 5.5. After filtration and drying of the precipitation, as-prepared Ni-decorated CNTs’ (abbreviated as Ni-CNTs) powders were obtained.

### 2.2. Preparation of Composite Foams

Melamine foam (Outlet Technology Co., Ltd., Chengdu, China) with an open porosity of about 99.8%, pore diameter of 100–200 µm, and wire diameter of 6–9 μm becomes conductive through the chemical deposition of silver and acts as the cathode in the subsequent composite electrodeposition. The composition of the electroplating solution was CuSO_4_·5H_2_O (200 g/L), H_2_SO_4_ (60 g/L), polyethylene glycol (0.03 g/L), 2-butyne-1,4-diol (0.2 g/L), and Ni-CNTs (1g/L). Two copper plates were used as anodes, and silver-deposited melamine foam was used as the cathode. Electrodeposition was conducted at a current density of 2 A·dm^−2^ for 7 h to obtain a Ni-CNTs/Cu foam with a complete continuous coating. Subsequently, the foam was heated at 450 °C for 2 h in a reducing atmosphere (H_2_:N_2_ = 1:10 volume) and then heated to 750 °C for 1 h. The main purpose of the heat treatment is to remove the melamine template and generate a dense, smooth foam coating. For proper comparison, Cu foam and CNTs/Cu foam were also prepared using the same procedure.

### 2.3. Preparation of Bulk Composites

**Figure 1:**
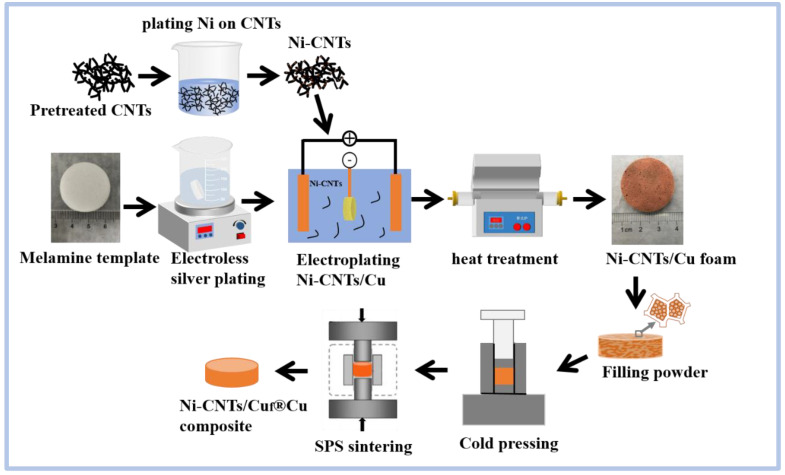
SA schematic illustration of the preparation process of the Ni-CNTs/Cu_f_^®^Cu composite.

Copper powder (40 µm, purity 99.9%, Shanghai Naiou Nanotechnology Co., Ltd., Shanghai, China) was filled into the pores of the foam until the top of the pore. Then, the composite was cold-compacted into a cylindrical block with a tablet press at a pressure of 10 MPa and a holding time of 1 min. The composite block was sintered using an SPS-20T-10 furnace. Sintering was conducted on an SPS furnace (SPS-LABOX-650F, SINTER LAND INC., Niigata, Japan) at 750 °C, for a holding time of 5 min, at a uniaxial pressure of 50 MPa under a vacuum of about 5 Pa. Figure 1 shows a diagram of the preparation process of the Ni-CNTs/Cu_f_^®^Cu composite.

For comparison, pure Cu, the Cu foam-reinforced Cu matrix composite (abbreviated as Cu_f_^®^Cu), and the CNTs/Cu foam-reinforced Cu matrix composite (abbreviated as CNTs/Cu_f_^®^Cu) were prepared with the same sintering parameters. The specific sample names and related parameters are shown in Table 1.

### 2.4. Characterizations

A field-emission scanning electron microscope (SEM, Nova Nano-450, FEI, USA) was used to characterize the morphology of the composite metal foams and fracture surfaces of composites. A microscopic laser Raman spectrometer (LABRAM HR800, HORIBA, NJ, USA) was used to examine the crystal structure information of the CNTs during processing. A high-resolution transmission electron microscopy (HRTEM, Tecnai G2-TF30 S-Twin, FEI, Portland, OR, USA) was used to examine the detailed microstructures of the Ni-CNTs’ powders and composites. The CNTs’ content of the composite foams was tested by a carbon flow analyzer (SDF2-HCS878, Beijing Hifid Technology Co., Ltd., China). Vickers microhardness was measured using an MC010 microhardness analysis system (Shanghai Micro Light-Machine Tech Co., Ltd., Shanghai, China) with a load of 0.98 N, and applied for 15 s. The electrical conductivity of the samples was measured by a direct-current low-resistance test instrument (Sigma 2008B, Xiamen Tianyan Instrument Co., Ltd., Xiamen, China). The mechanical properties of the pure copper and composites were tested with a universal testing machine (AG-X-100kN, SHIMADZU, Kyoto, Japan) at a crosshead speed of 0.2 mm·min^−1^.

## 3. Results and Discussion 

### 3.1. Characterization of Ni-CNTs/Cu Composite Foams

**Figure 2:**
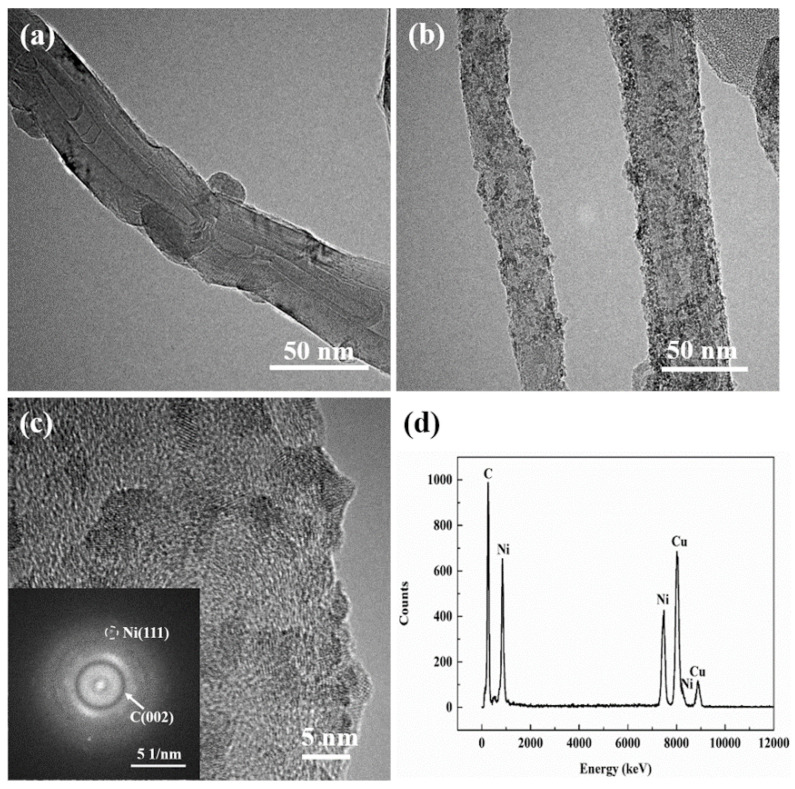
(**a**) TEM observation of raw CNTs; (**b**,**c**) TEM observation of Ni nanoparticles decorated CNTs; (**d**) EDX spectrum of Ni-CNTs.

The surface topography of raw CNTs is shown in Figure 2a. It can be observed that the raw CNTs have clear and smooth surfaces, regular walls, and hollow channels. Figure 2b,c show the TEM images of Ni-CNTs, which reveal the uniform distribution of nickel nanoparticles, with a mean grain size of about 3–5 nm, on the surface of the CNTs. Figure 2d is the EDX spectrum of Ni-CNTs; appearing in the graph are carbon peaks, nickel peaks, and copper peaks. Since the TEM observation of Ni-CNTs’ powder requires the use of a small-hole microgrid copper mesh, there are obvious copper peaks in the figure. The results show that the nanoparticles on the surface of CNTs are mainly composed of Ni. Ni deposition can reduce the surface energy of CNTs and prevent their agglomeration, while also improving the wettability and interfacial compatibility between CNTs and the copper matrix. Moreover, the copper matrix will form an infinite solid solution with the decorated Ni nanoparticles, and the interfacial bonding between the CNTs and Cu matrix will transform into infiltration bonding with stronger interfacial adhesion strength [25].

**Figure 3:**
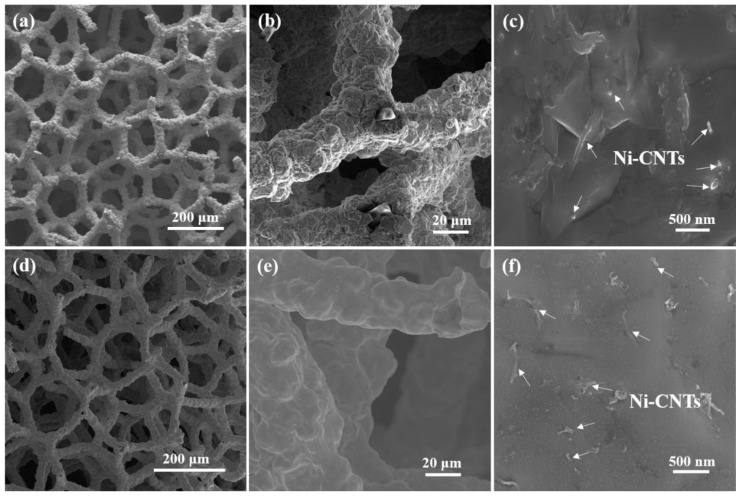
SEM images of Ni-CNTs/Cu foam before and after heat treatment at different magnifications; (**a**–**c**) before heat treatments; (**d**–**f**) after heat treatments.

Figure 3 shows SEM images of the Ni-CNTs/Cu foams at various magnifications before and after heat treatment. The three-dimensional network structure and dense coating surface of the open-cell composite foams can be clearly observed. As shown in Figure 3a,b the melamine template exists in the foam skeleton before heat treatment, and the melamine template has been removed after heat treatment. As shown in Figure 3d,e the foam has clear and non-clogged pores, and a dense and smooth coating. In Figure 3c–f, the Ni-CNTs are uniformly embedded in the foam skeleton without obvious agglomeration, inherited from the composite electrodeposition progress. Embedded CNTs are believed to benefit load transfer efficiency and improve mechanical function [26].

**Figure 4:**
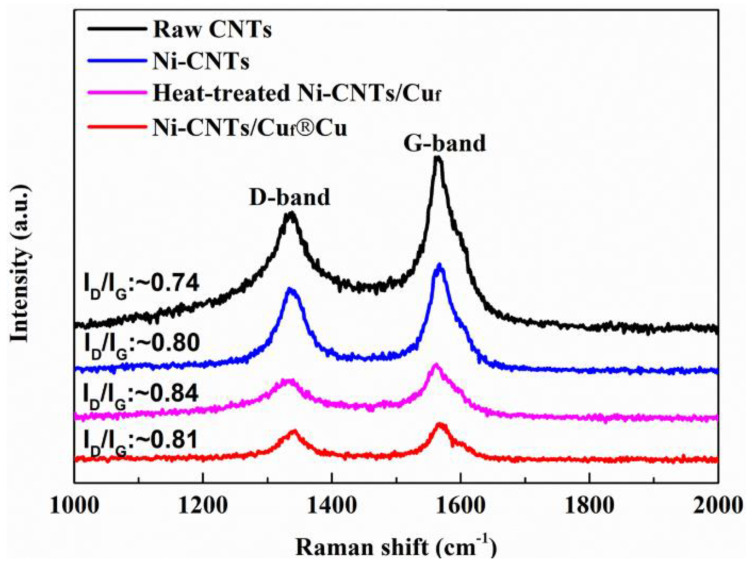
Raman spectra of raw CNTs, Ni-CNTs, and Ni-CNTs/Cu_f_ after heat treatment and Ni-CNTs/Cu_f_^®^Cu.

Figure 4 shows the Raman spectra of raw CNTs, Ni-CNTs, heat-treated Ni-CNTs/Cu_f_, and Ni-CNTs/Cu_f_^®^Cu samples. The intensity ratio of the D peak and G peak (I_D_/I_G_) can indicate the degree of graphitization of the carbon material. The I_D_/I_G_ ratios of the Ni-CNTs (untreated, heat-treated, and sintered) were determined to be approximately 0.80, 0.84, and 0.81, respectively. The similarity between these values indicates that the heat treatment and SPS sintering process caused little structural damage to the CNTs.

### 3.2. Electrical Conductivity

**Figure 5:**
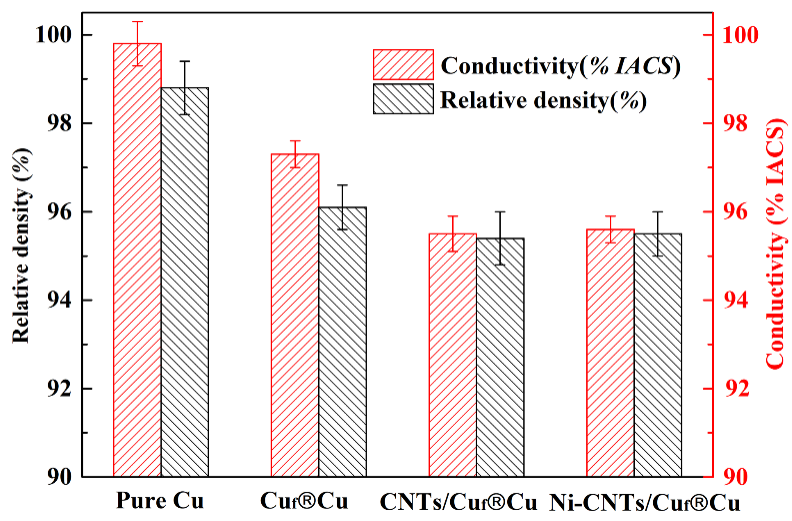
Electrical conductivity and relative density of pure Cu and the composites.

In this paper, electrical conductivity is expressed by the International Annealed Copper Standard (IACS). As shown in Figure 5, the Cu_f_^®^Cu composite possesses a conductivity of 97.3% IACS, which is very close to the pure, SPS-sintered Cu sample (99.8% IACS). The conductivities of the CNTs/Cu_f_^®^Cu and Ni-CNTs/Cu_f_^®^Cu composites were 95.5% IACS and 95.6% IACS, respectively, slightly lower than that of pure Cu. The additions of CNTs or Ni-CNTs in the composites is as low as 0.04 wt.%, the continuity between copper grains is high, and the possibility of electron scattering is small; thus, the conductivity of the composites shows little reduction [27]. In other reports, it has been shown that mechanical strengths are significantly improved by coating CNTs with Ni, but the electrical conductivity of the composite shows a downward trend [21,28]. However, in this study, the CNTs are only distributed in the skeleton phase, and the CNTs or Ni-CNTs content is much lower compared to regular CNTs-reinforced Cu composites. The continuous pure copper phase distributed in the foam skeleton pores contributes highly to the electrical conductivity; thus, the addition of CNTs and the nickel decoration have caused only slight decreases in the electrical conductivity.

### 3.3. Mechanical Properties 

**Figure 6:**
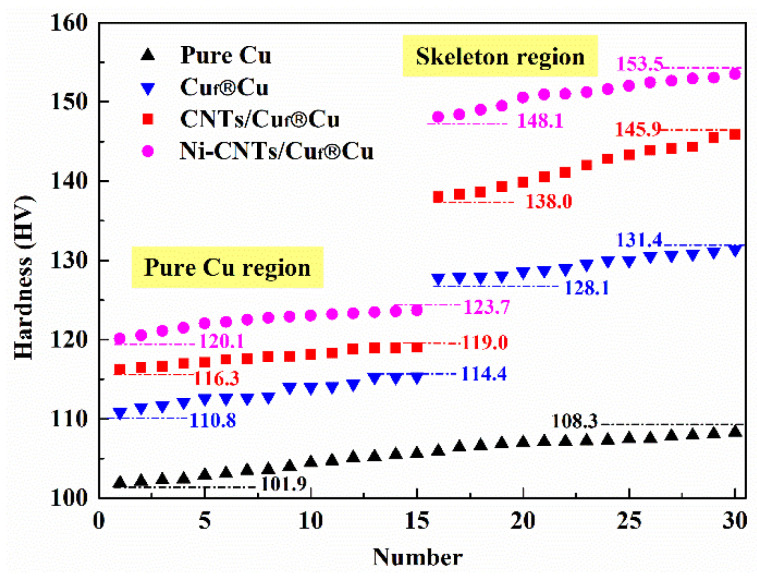
Vickers microhardness of pure Cu and composite samples.

Since the reinforcing phase and matrix phase of a composite form a network interpenetrating structure in three-dimensional space, its hardness zone can be divided into the foam skeleton area and pure copper area. As shown in Figure 6, the hardness value of pure Cu is 101.9–108.3 HV, while the hardness values of the Cu_f_^®^Cu pure copper area and composite skeleton area are 110.8–114.4 HV and 128.1–131.4 HV, respectively. This indicates that the three-dimensional skeleton structure of the Cu foam with smaller grain size is retained after SPS sintering. The hardness value shows a linear increase with the additions of CNTs and Ni-CNTs. Compared with the CNTs/Cu_f_^®^Cu composite, the microhardness of the Ni-CNTs/Cu_f_^®^Cu composite has significantly improved, which is 120.1–123.7 HV for the pure copper area and 148.1–153.5 HV for the skeleton area. The increased hardness value of the pure copper area may be related to the SPS sintering process. SPS uses uniaxial pressure and pulses a direct current to heat and sinter the samples. During the SPS sintering process, the conductivity changes of the samples and the different pressure distributions of the copper powder will affect both the sintered neck growth and copper’s grain growth [18]. The increased hardness of the skeleton area can be attributed to the changes in its interface microstructure and surface coating, which provide stronger interfacial bonding to facilitate load transfer [29]. Therefore, a higher hardness value is achieved in Ni-CNTs/Cu_f_^®^Cu, with the same weight fraction, as compared to the CNTs/Cu_f_^®^Cu composite.

**Figure 7:**
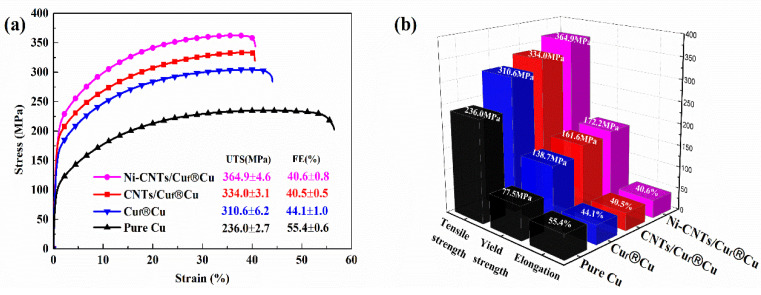
(**a**,**b**) The tensile stress–strain curves of pure Cu and composites.

Figure 7 shows the tensile stress–strain curves of the SPS-sintered samples. All of the foam-reinforced composites show obvious improvement in tensile strength as compared to the pure copper sample, and the tensile elongations maintain a high value of about 40%. This indicates that the composite structure design with skeletons as three-dimensional reinforcements offers an effective way to overcome the paradox between strength and ductility in composites. The tensile strength of Ni-CNTs/Cu_f_^®^Cu is about 364.9 MPa, significantly higher than CNTs/Cu_f_^®^Cu (~334.0 Mpa), and this is possibly due to the improved interfacial bonding through nickel decoration. The Ni-CNTs help improve the anti-deformation ability of Cu foam and enhance the interfacial bonding ability of CNTs and Cu. It is worth noting that the CNTs in this experiment are only uniformly dispersed through the metal foam skeleton and not through the entire composite. Table 2 summarizes the tensile properties and electrical conductivity of the CNTs/Cu composites in this study and the literature. From the statistical results, it can be clearly observed that interface improvement is an effective way to improve the mechanical properties of CNTs/Cu composites. The electrodeposition process in this experiment is beneficial for improving the dispersibility of CNTs, and the formation of interfacial carbides facilitates the transformation of weak interfacial bonding between CNTs and Cu into strong interfacial bonding. Therefore, the load transfer ability of Ni-CNTs during deformation can be utilized to improve the strengthening efficiency of CNTs. Compared with traditional CNTs/Cu composites prepared by powder metallurgy [22,30,31], the CNTs/Cu_f_^®^Cu and Ni-CNTs/Cu_f_^®^Cu composites prepared in this study possess both high-strength ductility and excellent conductivity with much lower CNTs content. The uniformly distributed reinforcements, good interfacial bonding, and network interpenetrating three-dimensional reinforcing skeleton are the main reasons for this unique combination of high strength, satisfactory plasticity, and good conductivity.

**Figure 8:**
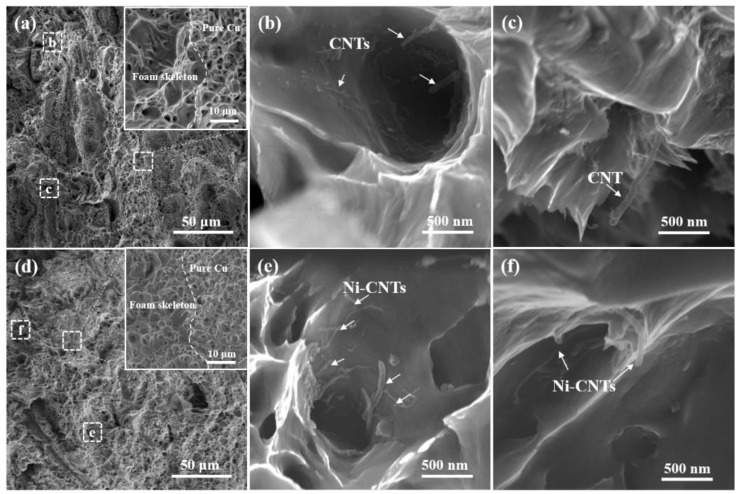
Fracture morphology observation: (**a**) CNTs/Cu_f_^®^Cu composite; (**b**,**c**) enlarged images in the corresponding area of (**a**); (**d**) Ni-CNTs/Cu_f_^®^Cu composite; (**e**,**f**) enlarged images in the corresponding area of (**d**). The inset shows the magnified images of the selected areas.

Figure 8 shows the fracture morphologies of the CNTs/Cu_f_^®^Cu and Ni-CNTs/Cu_f_^®^Cu composites. From Figure 8a–d, it is observed that there are two different regions in the microscopic fracture morphology of the composite. The dimple size and depth in the area on the right are relatively uniform, with no CNTs, and it is determined to be the pure Cu area, while the foam skeleton area is relatively shallow and contains uneven dimples. It can be observed from the shape of the dimples that the plasticity of the skeleton area is relatively poorer than that of the pure copper area. In comparison, the well-distributed dimples in Figure 8d indicate that Ni nanoparticles on the surface of CNTs help improve the dispersion effect of CNTs during electrodeposition. Figure 8b,c show the fracture surface in the skeleton area with ductile fracture surfaces, sharp tail dimples, and CNTs. As shown in Figure 8e,f, the well-dispersed embedded Ni-CNTs in the dimples indicate that the interface between the Ni-CNTs and Cu is very firmly bonded. CNTs are embedded in the metal matrix and held at one end, depending upon the bonding energy and extent of the embedded portion [32,33]. Due to the surface modification of the CNTs, the interface bonding between the CNTs and Cu matrix is enhanced so that the Ni-CNTs bear more load and lead to improved composite strength.

**Table 2:** Tensile properties and conductivities of composites prepared in this study and the literature.

Materials	CNTs Content	Conductivity (%IACS)	UTS (MPa)	FE (%)	Ref.
Ni-CNTs/Cu_f_^®^Cu	0.04 wt.%	95.6	364.9	40.6	This work
CNTs/Cu_f_^®^Cu	0.04 wt.%	95.5	334	40.5	This work
CNTs/Cu–Ti	0.4 wt.%	--	355	22.8	[22]
TiC@CNTs/Cu	1.5 vol.%	87.5	281.0	20.1	[23]
Ni-CNTs/Cu	0.5 vol%	93	292	34	[25]
Cu/CNTs	0.5 vol.%	92.9	275	24	[30]
Cu@CNTs/Cu	0.4 wt.%	93.6	272	14.3	[33]

### 3.4. Interfacial Microstructure

**Figure 9:**
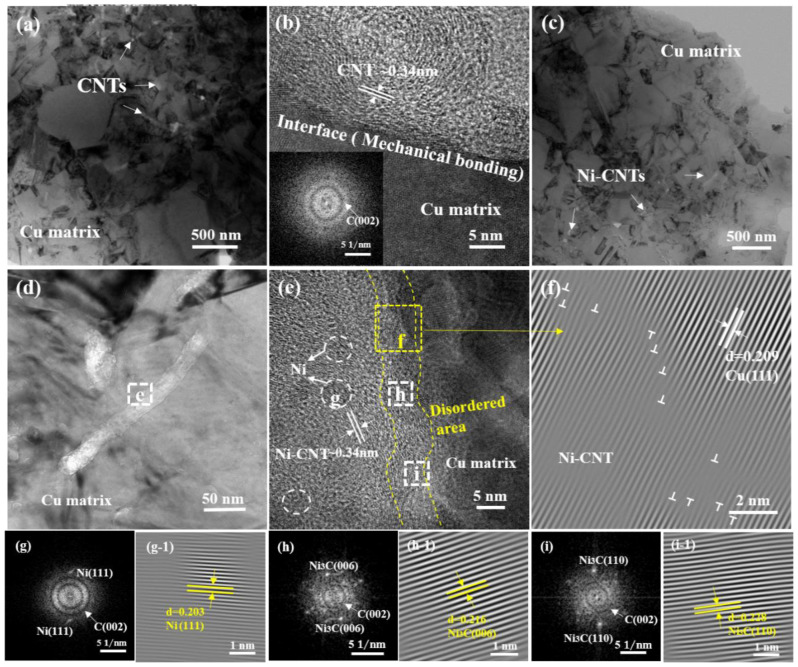
(**a**,**b**) TEM images of CNTs/Cu_f_^®^Cu composite; (**c**–**e**) TEM images of Ni-CNTs/Cu_f_^®^Cu composite; (**f**–**i**) the corresponding image in the rectangular box of (**e**).

To a large extent, the performance of CNT-reinforced copper composites depends on the interfacial bonding strength. Figure 9 shows a TEM observation of the interface of the CNTs/Cu_f_^®^Cu and Ni-CNTs/Cu_f_^®^Cu composite. As shown in Figure 9a–c, two regions with obvious differences in grain size are observed. The area with the larger grain size is the pure copper area, and the area with the smaller size is the skeleton area in which CNTs are uniformly dispersed without agglomeration. As shown in Figure 9b, the interfacial transition between the graphite-structured CNT and the Cu matrix at the interface is very sharp and abrupt. Combined with the inserted Fast Fourier Transform (FFT) image, the outer wall of the CNT still maintains a graphitic structure with good crystallinity, and the interfacial bonding is very smooth. This is a typical mechanical bonding interface. Generally, there is little wettability between Cu and C, so this interface is considered to have weak interfacial bonding force [31]. As shown in Figure 9e, a disordered area can be observed between the Cu matrix and the Ni-CNTs, and the interfacial boundary between Cu and Ni-CNTs is not as sharp as the mechanical bonding interface in the CNTs/Cu_f_^®^Cu composite. Figure 9f shows the corresponding inverse fast Fourier transform (IFFT) image of area f in Figure 9e. There is no interfacial gap between the Ni-CNTs and the Cu matrix. Several accumulated dislocations were observed in the Cu matrix near the interfaces, indicating that Ni-CNTs can effectively hinder the movements of dislocations. The dislocations at the interface were shown to promote the interfacial bonding between CNTs and the Cu matrix [34]. Figure 9g–i show a TEM image of the Ni-CNTs/Cu_f_^®^Cu composite and the corresponding FFT and IFFT. Ni nanoparticles can be observed on the surface of the CNTs, such as the zone marked by the yellow dotted line in Figure 9e. The plane spacing of the lattice fringe is 0.203 nm, which corresponds to the (111) plane of Ni (Figure 9g). The interface between the Ni-CNT and Cu becomes very distorted, which indicates that the inter-diffusion between C and Cu/Ni atoms leads to the formation of the interfacial transition region [31]. The d orbital of Ni and the p orbital of the CNTs can form a hybrid; thus, there is strong chemical adsorption and interfacial bonding between Ni and the CNTs [35]. In addition, Cu and Ni have the same face centered cubic structure and can form solid solutions in any proportion, which will enhance the metallurgical bonding [36]. As shown in Figure 9h,i, it can be found that the metastable Ni3C is formed at the interface, and these nano-crystals are unevenly distributed and tend to be in the defect sites on the CNT’s surface. Combined with the non-equilibrium sintering process of SPS, the reaction between the functionalized CNT and the Ni can form a strong chemical bond. Simultaneously, the in situ-formed Ni3C at the interface could be regarded as the interphase between the CNTs and the Cu matrix [37]. These interphases can link the CNTs at the defect sites and act as bridges between CNTs and the Cu matrix, resulting in high load-transfer efficiency. The formed transition zone not only provides the source of initial dislocations but also serves as a large-capacity area for dislocation pinning and accumulation during deformation, thereby forming an interface with a high load-bearing capacity [37,38].

### 3.5. Strengthening Mechanism

In CNTs/Cu composites, various reinforcement mechanisms can be considered: grain refinement [33], load transfer strengthening [6], and dislocation-strengthening mechanism [39]. In this study, CNTs have a large aspect ratio, regular arrangement, and low content, and the Orowan strengthening effect is negligible [40]. A combination of grain refinement and load transfer was reported to be the possible strengthening mechanism in CNTs/Cu composites [33,38,41].

It is worth noting that the electrodeposited composite foam has a smaller grain size, and the addition of Ni-CNTs or CNTs further refines the grains. To facilitate the discussion of the influence of the interfacial strength on the material properties, the Cu_f_^®^Cu sample is used as the matrix in this experiment. The improved strength via the grain refinement-strengthening mechanism in composites can be estimated from the Hall–Petch equation:

(1) $$\Delta \sigma_{GR}=K\left(d^{-\frac{1}{2}}-d_{0}^{-\frac{1}{2}}\right)$$

where K is a constant (0.14 $\mathrm{MPa}\cdot m^{\frac{1}{2}}$ for Cu) [42], and d and d_0_ are the average grain size of the composite and Cu_f_®Cu sample, respectively.

**Figure 10:**
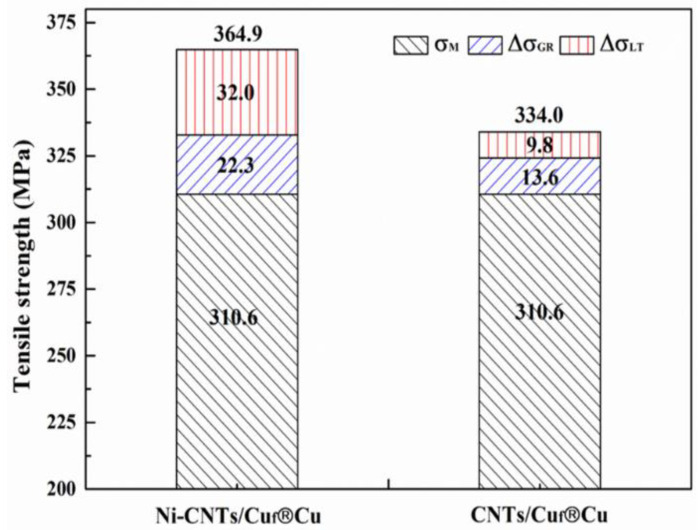
Strengthening factors in composites.

If we assume the tensile strength of a composite, then ($\sigma_{c}$) could be regarded as the summation of the corresponding pure Cu_f_^®^Cu strength ($\sigma_{M}$), grain refinement ($\Delta \sigma_{GR}$), and load transfer ($\Delta \sigma_{LT}$), then $=-\sigma_{M}-\Delta \sigma_{GR}$. The obtained strengthening factors contributing to $\sigma_{c}$ are summarized in Figure 10. Compared with the CNTs/Cu_f_^®^Cu composite, the load transfer of CNTs is a major contributor to the total strength. By modifying the surface of CNTs, the load transfer of the Ni-CNTs/Cu_f_^®^Cu composite increased from 9.8 MPa to 32.0 Mpa, which is an increase of 226.5%. This indicates that the partial reaction of CNTs with the interface results in a better load-transfer effect. Duan [21] demonstrated the pull-out process of Ni-CNTs from the Cu matrix based on the viewpoint of molecular dynamics, and the results showed that the interfacial strength was significantly improved after coating Ni on CNTs. Their research results are consistent with the experimental results of this paper, which shows that, by modifying the CNT surface, a CNTs/Cu composite with high interfacial bonding strength can be obtained.

## 4. Conclusions

In this study, a Ni-CNTs/Cu composite foam was used to reinforce copper-based composite materials. By modifying CNTs with Ni nanoparticles, a nanoscale interfacial transition region with high carrying capacity and tight interfacial bonding is obtained, which effectively improves the interfacial bonding strength of CNTs and Cu. The experimental results show that the Ni-CNTs/Cu_f_^®^Cu composite has a tensile strength of 364.9 MPa, which is about 54.6% higher than the pure Cu samples. The main enhancement mechanism is fine grain enhancement. In addition, the electrical conductivity and elongation of the composite are maintained at an excellent level of 95.6% IACS and 40.6%. The Ni-CNTs/Cu_f_^®^Cu have excellent comprehensive properties, which are mainly attributed to the introduced three-dimensional skeleton network and the solid interfacial bonding of the uniformly embedded CNTs and Cu. Therefore, using Ni-CNTs/Cu foam with an optimized interface as a reinforcement is beneficial for obtaining a unique combination of high strength, plasticity, and electrical conductivity, improving the overall performance of the composites. This new idea provides guidance for the preparation of copper-based composites with excellent electrical conductivity and improved mechanical properties.

## Figures and Tables

**Table 1:** The sample names and related parameters.

Sample	Reinforcing Phase	Foam Porosity (%)	CNTs in Foam (wt.%)	CNTs in Composite (wt.%)
Pure Cu	--	--	0	0
Cu_f_^®^Cu	Cu foam	90.89	0	0
CNTs/Cu_f_^®^Cu	CNTs/Cu foam	90.61	0.16	0.04
Ni-CNTs/Cu_f_^®^Cu	Ni-CNTs/Cu foam	90.54	0.16	0.04

## Data Availability

The datasets used or analyzed during the current study are available from the corresponding author upon reasonable request.
